# Variation among populations of *Quercus macrocarpa* in a reciprocal transplant experiment across a latitudinal gradient

**DOI:** 10.1002/ecy.70517

**Published:** 2026-10-04

**Authors:** Lucy M. S. Rea, Laura Ostrowsky, Rebekah Mohn, Mira Garner, Lindsey Worcester, Cathleen Lapadat, Heather R. McCarthy, Andrew L. Hipp, Jeannine Cavender‐Bares

**Affiliations:** ^1^ Department of Ecology, Evolution, and Behavior, College of Biological Sciences University of Minnesota St Paul Minnesota USA; ^2^ Department of Organismic and Evolutionary Biology Harvard University Cambridge Massachusetts USA; ^3^ Herbarium and Center for Tree Science The Morton Arboretum Lisle Illinois USA; ^4^ Pritzker Laboratory for Molecular Systematics and Evolution The Field Museum Chicago Illinois USA; ^5^ School of Biological Sciences University of Oklahoma Norman Oklahoma USA

**Keywords:** local adaptation, plasticity, *Quercus macrocarpa*, reciprocal transplant, tree ecophysiology

## Abstract

Ongoing climate change will negatively impact tree populations unless they are able to acclimate to the changes in their local environment. Effective planning for climate adaptation management requires an understanding of the current state of local adaptation and physiological performance to assess whether populations are at risk of local extinction, to determine if seed movement is appropriate, and to select appropriate seed sources if intervention is needed. We established a new reciprocal transplant experiment (Adaptation to Climate and Environment [ACE]) across a latitudinal gradient in North America spanning a range of climate and soil conditions to examine the impacts of warming on three bur oak (*Quercus macrocarpa*) populations across much of the species' range. We established common gardens in Minnesota, Illinois, and Oklahoma in 2021 grown from seeds collected in 2018–2019 within 50 km of each of those locations from a total of 60 maternal families. In 2023, we measured physiology and 3‐year growth and survival to understand local adaptation in bur oak populations. We aimed to (1) assess local adaptation in each of the populations using survival and size as fitness metrics and (2) evaluate physiological responses to different environments along the latitudinal gradient. We found evidence for local adaptation of the southern population in the southernmost garden, but that the northern populations were maladapted to high levels of warming, as evidenced by their low survival, growth, and photosynthetic rates in the warmest common garden. However, mild climate and relatively high nutrient conditions in the middle garden resulted in the highest fitness and best physiological performance for all populations. Growth and survival were correlated in the middle garden but were decoupled in the northern and southern gardens, likely due to stress associated with more extreme climates at the ends of the gradient. Our results suggest that southern seed sources may perform well in warmer conditions in the north brought on by climate change, which has important implications for managers assisting broadly ranged tree species in adapting to climate change.

## INTRODUCTION

Warming temperatures associated with climate change will have implications for the long‐term persistence of locally adapted populations as they may experience adaptational lags that cause them to become maladapted to the novel climate conditions and may perform better at higher latitudes outside their historic range (Browne et al., 2019; Etterson et al., 2020; Gorton et al., 2022; Sáenz‐Romero et al., 2017). Populations that have higher fitness in their local environment than foreign populations or higher fitness in their home environment than away environments are said to be locally adapted (Kawecki & Ebert, 2004; Williams, 1966). Local adaptation in large populations of widespread species can help maintain genetic variation within the species, which is critical in species' persistence through changing climatic conditions, and understanding local adaptation can provide useful information that can help improve adaptative potential of populations not currently at high risk of extinction (Meek et al., 2023; Whitlock, 2015).

Trees face major threats from rapid climate change and may be particularly at risk due to their long generation time that limits their capacity to adapt quickly (Aitken et al., 2008). Given that forests are estimated to be the largest terrestrial carbon sink on the planet, reforestation is one of the most effective carbon drawdown solutions proposed, and it is especially critical to avoid losses of tree species and their genetic diversity, which provides the basis for adaptation to novel environments (Bastin et al., 2019; Joshi et al., 2001; Pan et al., 2024). Models predict that habitat suitable for temperate forests will expand northward (Rehfeldt et al., 2012); however, the distance that seed and pollen can move may slow the rate at which trees' ranges are able to expand. Dispersal limitations on the movement of tree species will necessitate population‐level responses to novel climates through phenotypic plasticity or adaptation (Franks et al., 2014; Hoffmann & Sgrò, 2011; Huntley, 1989). Adaptational lags coupled with dispersal limitations may necessitate anthropogenic intervention to ensure survival of populations that cannot tolerate novel climate regimes in their current environment. Often, locally adapted genotypes are selected for planting in reforestation and regeneration efforts; however, shifting climates may undercut the common assumption of “local is best” (Bower et al., 2024; Joshi et al., 2001; Pike et al., 2020). Seed transfer (also referred to as population assisted migration), the practice of moving populations within their existing species range, may support locally adapted populations that have become maladapted to their current environment due to climate change. Many foresters anticipate the need for seed transfer plantings in the near future that improve their forests' ability to persist through climate change impacts (Clark et al., 2024). Movement of seeds can increase genetic diversity and the probability of survival in changing environments. However, data are needed to understand which populations and genotypes may be maladapted to climate change, and which have adaptations suited to novel conditions brought on by climate change (Xu & Prescott, 2024).

Local adaptation can be tested with reciprocal transplant experiments, which separate genetic from environmental (plastic) influences on phenotypes (Kawecki & Ebert, 2004; Schwinning et al., 2022). We established a reciprocal transplant experiment using the deciduous, broad‐leaved tree species *Quercus macrocarpa* Michx. across a latitudinal gradient by collecting seeds from maternal families across the gradient to establish the Adaptation to Climate and Environment (ACE) experiment. *Q. macrocarpa* is a useful species for such an experiment because it has a broad latitudinal and ecological range. Consequently, it is likely to show adaptive differentiation across its range of contrasting climatic conditions. *Q. macrocarpa* is also an important species in oak savannas, a rare and threatened ecosystem, due to its fire resistance capabilities (Hoekstra et al., 2005). Furthermore, *Quercus* species are ecologically important tree species supporting wildlife diversity as they are an important food source for birds, insects, and mammals (Tallamy, 2021). *Quercus* species are also relevant in a management context, as they are the most common genera planted in adaptation plantings by forest managers (Clark et al., 2024). By understanding local adaptation in populations of wide‐ranging tree species, we can provide valuable information on seed sourcing for land managers in seed transfer efforts, which can be a critical strategy in helping to prevent species extinction and economic losses and to maintain ecosystem services (Abrams et al., 2021; Krech et al., 2004; Williams & Dumroese, 2013). Our goals are to understand the underlying mechanisms of local adaptation in *Q. macrocarpa* populations by taking a novel approach to integrate ecophysiology, spectral reflectance, and fitness. We do this by measuring high‐throughput ecophysiological traits and processes that provide insight into the performance of our three populations in different environments and to combine survival and growth in a single measure of fitness using aster models (Geyer et al., 2007; Shaw et al., 2008).

In this study, we tested for local adaptation and adaptive differentiation by reciprocally transplanting seedlings from three populations in field conditions. We collected acorns from individual trees (half‐sib families) within three populations (Minnesota, Illinois, and Oklahoma) across a climatic gradient which spans a mean annual temperature (MAT) gradient of 6–16°C. ACE was designed to reciprocally plant seedlings from each family within each population into gardens in those three states using a quantitative genetics design, such that seedlings from all families were randomized in replicated blocks in each garden. We addressed the overarching question: Do *Q. macrocarpa* progeny from three bur oak tree populations have higher fitness and physiological performance in the garden closest to their origin, or are they better adapted to a climate that has shifted northward due to warming with climate change? If populations are locally adapted, we expect them to have higher fitness and adaptive physiological performance when they are in their home garden (home vs. away), and to have higher fitness than other populations in their home garden (local vs. foreign) (Appendix S1: Figure S1). On the other hand, if populations are adapted to a climate that has shifted northward due to the 1.1°C warming in the past century (IPCC, 2023), we expect them to have higher fitness and physiological performance in the more northern (higher latitude) gardens compared to the hotter and drier southern (lower latitude) gardens, but northern populations to exhibit lower fitness and higher physiological stress responses in the southern garden. Furthermore, we sought to understand mechanisms behind differences in populations' fitness in different environments by tracking differences in physiological responses and phenological timing. We expect that the physiological responses will demonstrate adaptive differentiation by providing evidence for genetic differences among populations in functional traits that are advantageous in their home environment. We expect that genotypes and populations from the south where growing seasons are longer and cold stress is reduced will exhibit physiological traits aligned with extended phenology that confers longer leaf life spans (Chabot & Hicks, 1982; Kikuzawa, 1991; Wright et al., 2004). We also anticipate that these southern genotypes will maintain productivity through hot, arid conditions (Oram et al., 2023). In contrast, in populations from higher latitudes with shorter growing seasons we expect genotypes to have higher photosynthetic rates and higher relative growth rates (RGRs) (see Appendix S1: Figure S2). Higher carbon assimilation rates are expected to compensate for the shorter growing season, although allocation of photosynthate to cold tolerance or functions other than growth could lead to decoupling of photosynthesis and growth rates (Raven, 1997; Bosiö et al., 2014; MacArthur, 1984). We thus expect to see a tradeoff between growth and survival in more stressful gardens, as carbon resources from photosynthesis are allocated to tolerating stresses as opposed to growth. Because seedling transplants were grown in natural field conditions that are experiencing ongoing climate change, there may be edaphic differences among sites that can offer more favorable soil resource conditions at some sites relative to others and complicate interpretations of local adaptation in relation to climate.

## METHODS

### Reciprocal transplant experimental design

The Adaptation to Climate and Environment (ACE) bur oak experiment was designed to test for local adaptation by reciprocally transplanting *Quercus macrocarpa* seedlings from known families into three garden sites across a latitudinal gradient. The design was based on prior work from Deacon and Cavender‐Bares (2015), Etterson (2004a, 2004b), Etterson and Shaw (2001), and Ramírez‐Valiente et al. (2018). Acorns from Minnesota, Illinois, and Oklahoma populations were collected in 2018 and 2019 from individual mother trees at least 30 m apart to minimize the likelihood of shared pollen among trees (see Rea et al., 2026b, supplemental material fig. S1 for map of collections). Acorns collected from mothers at each site (e.g., park, forest preserve, or natural area) were treated as a single subpopulation. Both 2018 and 2019 collections were germinated outdoors in the ground in a common environment in Vallonia, Indiana, at the Vallonia Nursery through the Indiana Department of Natural Resources, grouped by mother tree (see Appendix S1: Section S3, Methods supplement for supplemental details on acorn processing). In March 2021, seedlings were lifted from the common nursery, sorted and labeled by mother tree, and sent to be planted in the common gardens in tilled former grasslands and old fields in Minnesota (Cedar Creek Ecosystem Science Reserve, East Bethel, MN), Illinois (Morton Arboretum, Lisle, IL), and Oklahoma (Kessler Atmospheric and Ecological Field Station, Purcell, OK). The bare root seedlings were stored in cold storage until the weather was appropriate for planting (Planting times: Oklahoma—March, Illinois—April, Minnesota—May).

In each of the common gardens we planted 10 individuals from each of the 20 families from provenances in Minnesota, Illinois, and Oklahoma, which are grouped into populations named by their provenance. The seedlings were planted 1 m apart in a randomized block design, with at least one individual from each family planted in each block (Figure 1a). Large herbivores were excluded with fencing around the perimeter of the gardens, and cages were placed around the seedlings to avoid rodent damage. Gardens were weeded or mowed through each growing season and mulched to suppress herbaceous understory growth. Due to symptoms of Phytophthora root rot that were detected in the summer of 2021, SUBDUE GR fungicide (a granular fungicide) was applied to all of the seedlings in the fall of 2021. The seedlings were irrigated for the first 2 years after planting to ensure the gardens were established (see Appendix S1: Section S3, Methods supplement for detailed information on garden maintenance).

**FIGURE 1:**
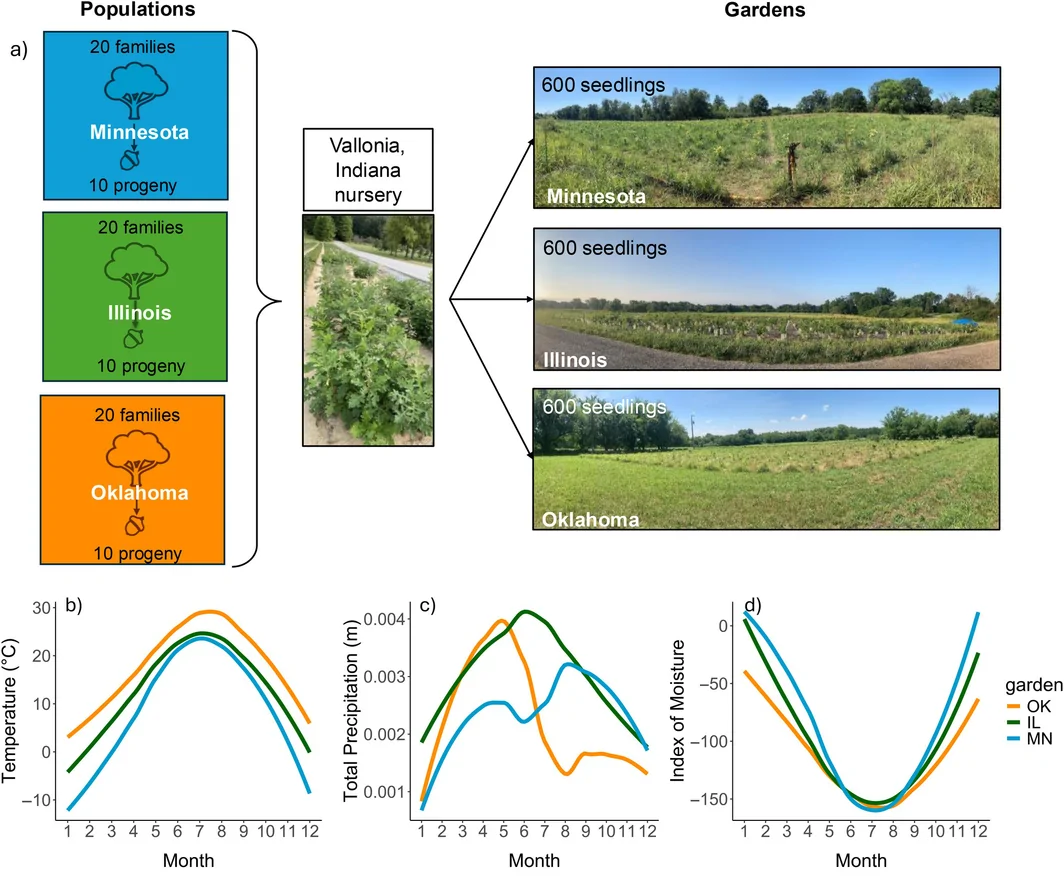
Reciprocal transplant garden design and climate information. In the top panel (a), the experimental design of the Adaptation to Climate and Environment (ACE) bur oak experiment is depicted. Three common gardens are located across a latitudinal gradient in Minnesota (MN, Cedar Creek Ecosystem Science Reserve), Illinois (IL, Morton Arboretum), and Oklahoma (OK, Kessler Field Station). In each garden, 600 seedlings were planted representing the three populations from Minnesota (blue), Illinois (green), and Oklahoma (orange). Each population comprised 20 half‐sibling maternal families, with half‐sibling acorn progeny collected from one known mother tree. Ten progeny from each family were planted in each garden. Photos of each site were taken in summer of 2022 after 1 year of planting. The “Populations” illustrations were created by Lucy M. S. Rea using icons of “Tree” and “Acorn” from Microsoft Powerpoint. Photo credits: Lucy M. S. Rea. In the bottom row, ERA5 monthly climate data averaged over the years 2021–2023 is shown for temperature (b), total precipitation (c), and the index of moisture (d). Colored lines indicate gardens: Minnesota (blue), Illinois (green), and Oklahoma (orange).

### Climate data

Monthly temperature data from 1940 to 2023 were acquired from ERA5 data (Hersbach et al., 2023) using garden coordinates, as weather station data were not consistently available for all sites for that time period. We extracted the ERA5 data from the raster file using QGIS (*QGIS Geographic Information System*, v3.34, QGIS.org, 2024). Temperature data for each year were averaged to calculate the MAT and were averaged over the months of May to September to calculate the growing season temperature for each year. To find the change in MAT and growing season temperature since the middle of the 21st century, we calculated the past mean for the years 1940–1970 and the recent mean for 1993–2023, then subtracted the past mean from the recent mean for each garden. We used a similar approach to extract precipitation data for the years of 2021–2023, while the garden was planted.

To estimate differences in aridity between the gardens, we calculated an index of moisture (IM) for each of the gardens following Ramírez‐Valiente & Cavender‐Bares (2017), where the IM is equal to the difference between monthly precipitation and monthly potential evapotranspiration (PET). The IM reflects the monthly water balance for an area and indicates water deficit with negative values and water surplus with positive values. We calculated PET with the Thornthwaite (1948) method, using monthly mean temperature and average day length per month.

### Soil nutrients and texture

To document differences in soil properties among sites, we sampled soil from the center of each garden block to 10 cm depth and conducted tests for soil nutrients (carbon‐nitrogen ratios [C:N], total nitrogen content) and soil texture. To measure soil nutrients, we dried the soil and sieved it with a 2‐mm mesh sieve to remove large rocks and organic matter. To measure the total soil carbon and nitrogen concentration, we ground the soil to a homogenous fine powder and analyzed it with a Costech ECS4010 elemental analyzer (Valencia, California, USA). We approximated the soil texture using the “texture by feel” method (Thien, 1979).

### Survival and growth surveys

To determine seedling performance and fitness, the seedlings were surveyed for survival and size after planting and yearly from 2021 to 2023 at the end of the growing season. The height measured from ground surface to the tip of the highest living bud and basal diameter measured with a caliper 5 cm from the ground were used as metrics of size. Stem volume (*V*) was estimated using a conoid equation as a proxy for aboveground biomass.

We used conoid volume (*V*):

(1) $$V=H\times \pi \times {D_{\mathrm{rc}}}^{2}/12,$$

where *H* is the height of the tree from the root collar to the apical meristem, and *D*_rc_ is stem diameter at the root collar.

RGR over the 3 years since the time of planting was calculated as follows:

(2) $$\mathrm{RGR}=\ln\left(V_{\text{final}}\right)-\ln\left(V_{\text{initial}}\right)/T_{\text{final}}-T_{\text{initial}},$$

where *V* is stem volume and *T* is time in total days. We calculated percent survival of maternal family groups in each garden as the number of trees alive divided by the number of trees originally planted in each family group.

### Phenology

In 2023, to determine growing season length, we tracked spring and fall phenology using protocols established by the National Phenology Network (Denny & Crimmins, 2023). In the spring, approximately weekly, we surveyed the trees in each garden and tracked if the trees were breaking leaf bud. Leaves were considered to be breaking leaf bud if a green leaf tip was visible at the end of the bud. At similar intervals in the fall, we tracked senescence as leaves with late‐season color that had fallen or had recently fallen.

We used the budburst and the senescence data to estimate the growing season length of canopy foliage persistence, or how long foliage was present on the trees in a growing season. We calculated this as the difference between the mean initial budburst date and the mean initial senescence date to find the total length of the canopy foliage growing season.

### Gas exchange

To measure photosynthesis rates and stomatal conductance, we conducted measurements of leaf‐level gas exchange on every living tree in each of the three gardens using a LI‐6800 Portable Photosynthesis System (LI‐COR, Lincoln, NE, USA) to determine CO_2_ assimilation (*A*) (see Appendix S1: Section S3, Methods supplement for supplemental details on settings and parameters used for measurement). These data were collected in both 2022 (with irrigation) and 2023 (after irrigation ceased) at similar points in the growing season and using the same settings and approach. Because *A* changes over the course of the day with stomatal closure, we found the maximum for *A* by excluding measurements after stomatal closure occurred by examining graphs of stomatal conductance to see where stomatal conductance dropped to near 0 (see Appendix S1: Figure S3 for cutoff times in each garden for each year).

### Spectral indices

We collected hyperspectral reflectance measurements at the leaf level on every living tree in each garden in 2022 and in 2023 to add additional, nondestructive metrics of chlorophyll content and nitrogen content. We measured leaf reflectance on the uppermost, fully expanded, undamaged leaf on each tree using a portable field spectrometer (SVC HR‐1024i; Spectra Vista) and a leaf clip with an internal light source (LC‐RP PRO; Spectra Vista) (see Appendix S1: Section S3, Methods supplement for supplemental details on spectral data and processing). Using the spectra, we calculated the chlorophyll normalized difference index (ChlNDI)

(3) $$\left(\frac{R750-R705}{R750+R705}\right),$$

and the normalized difference nitrogen index (NDNI)

(4) $$\left(\frac{\log\left(1/R1510\right)-\log\left(1/R1680\right)}{\log\left(1/R1510\right)+\log\left(1/R1680\right)}\right).$$

The ChlNDI is positively correlated with chlorophyll *a* content with minimal influence of chlorophyll fluorescence (Gitelson & Merzlyak, 1994). The NDNI is positively correlated with percent nitrogen of vegetation (Serrano et al., 2002).

### Statistical analysis

#### Aster modeling of fitness

We used aster models to estimate fitness for each population in each garden, using components of fitness expressed early in each tree's' lifecycle, given that the trees were seedlings and not yet reproductively mature at the time of measurement. Aster models estimate fitness integrated over multiple dependent components that contribute to an organism's fitness. The models avoid issues of nonindependence of normally distributed predictor variables by modeling the dependence of fitness components expressed later in life on earlier fitness components with different statistical distributions (Geyer et al., 2007; Shaw et al., 2008). Aster models thus can incorporate multiple components of fitness into a unified fitness estimate for each population, rather than having individual predictors of fitness.

We modeled fitness using survival to 2023 and height conditional to survival to the last year of measurements, as our models' nodes, depicted graphically as

(5) Planted→Survival to2023→Height in2023.

We chose height as the final fitness estimate in our model because it is a useful predictor of eventual fecundity and is dependent on survival (Cornelius, 1994; Gamache & Payette, 2004). Survival to 2023 was modeled with a binomial distribution, and height was modeled with a normal distribution. As predictors in the aster models, we included population (*P*), garden (*G*), and the interaction between them, as well as the height at the time of planting (*S*) as a covariate to account for variation in size that could influence fitness, as follows:

(6) F=P×G+S.

We ran the models using the aster() function in the R package aster (v. 1.3.5., Geyer, 2025). We used the anova() function to compare models with and without the interaction effect to test for significance of the interaction effect. Once we settled on the model (Equation 6), we obtained means and SEs for each population in each garden using the aster predict() function for a dummy dataset with an “average individual” representing each population‐garden combination.

#### Statistical testing of performance metrics

We evaluated differences in *A*_max_, ChlNDI, survival, and growth among and within gardens for all populations using ANOVAs followed by Tukey post hoc tests with a population by garden interaction effect. The number of individuals in each population in each garden varied due to survival and the number of measurements we were able to obtain (see Appendix S1: Table S1 for number of individuals included in each measure). We also tested relationships between photosynthetic physiology (*A*_max_ and ChlNDI) with fitness components (survival and growth) using linear models to understand the relationship between physiology and fitness components.

#### Environmental gradients

We conducted principal components analyses (PCA) to understand the environmental gradient captured by the gardens and quantify how the trees' performance aligns with the environmental gradient. As climate variables, we calculated minimum and maximum monthly average temperatures, the average growing season precipitation (as monthly averages of May–September precipitation), and minimum IM (as the minimum IM in May–September) for each garden. For soil variables, we included the percent nitrogen, percent carbon, and percent C:N ratio for each block in each garden. We centered and scaled the variables and used the prcomp() function to conduct the PCA. We extracted the PCA scores for PC1 and PC2 and evaluated their associations with performance metrics including *A*_max_, ChlNDI, NDNI, survival, growth, and fitness using linear models. We used generalized linear models for analysis of PCA scores with survival using the function glm() and the “logit” link function, given that the survival data are binomial. Model fit was evaluated using Akaike information criterion (AIC) (Akaike, 1973).

#### Soil effects on traits

To understand the factors driving variance within and among gardens, we evaluated the effects of different soil nutrient levels within and across gardens on traits. We used linear mixed models with the lmer() function of the lmerTest package (Kuznetsova et al., 2017) to understand the effect on traits (RGR, NDNI, ChlNDI) with percent soil nitrogen, percent soil carbon, and population as fixed effects, and block nested in garden as random effects. To model mortality, we used a generalized linear mixed‐effects model with the glmer() function, with the same effects as the linear mixed models, but modeled the binomial survival data with the “logit” link function.

## RESULTS

### Site climate differences

The ERA5 dataset showed that the average monthly temperature decreased latitudinally, with the Minnesota garden having the lowest average temperatures each month and Oklahoma having the highest (Figure 1b). Notably, the Oklahoma garden never had a monthly average below freezing (0°C), whereas the Minnesota and Illinois gardens did in the winter months. We found that since 1940, temperatures have increased at each of the garden sites. MAT has increased by over 1°C in each garden from averaged 30‐year periods of 1940–1970 to 1993–2023, and the amount of temperature change increased with latitude, with the greatest change in MAT occurring in the northernmost garden, Minnesota, and the lowest change in temperature in the southernmost garden, Oklahoma (Appendix S1: Table S2). Over the same period, the average growing season temperature has also increased. Like MAT, the greatest change in growing season temperature was in Minnesota. However, unlike MAT, the smallest change in growing season temperature was in Illinois, the middle garden.

The gardens also differed in precipitation and aridity over the year. The Oklahoma garden peaked in precipitation in the springtime around April and May (Figure 1c). The Minnesota garden also had a small peak in precipitation at that time; however, the highest monthly precipitation occurred in late summer for the Minnesota garden, around August and September. Precipitation in the Illinois garden peaked mid‐summer, in June and July, and was the highest precipitation of the year among the three gardens. The Illinois garden was also the least arid of the three gardens during the growing season, though the IM was negative for all the gardens, indicating a water deficit during the growing season (Figure 1d). Notably, the IM was negative year‐round for the Oklahoma garden, suggesting it was in an atmospheric water deficit for the entirety of the year while the gardens were planted, though some of the water stress was presumably alleviated by irrigation in the first 2 years of growth.

### Fitness components

We assessed two main components of fitness: survival and plant size. Survival rates of family groups were most disparate in the Oklahoma garden, where survival of the Minnesota population (MN) was significantly lower than the Illinois (IL) and Oklahoma (OK) populations (*F*(2, 181) = 8, *p* = 0.0004). The higher survival of OK compared to MN is in line with local versus foreign local adaptation (Figure 2a). Garden had a strong effect on survival percent (*F*(2, 181) = 34, *p* = 2.88e−13). Survival for MN and IL was highest overall in the Illinois garden. Survival was not significantly different across populations in the Illinois and Minnesota gardens. There was a significant population × garden interaction for survival, as survival differed across gardens, particularly for the IL and MN populations (*F*(4, 181) = 5.5, *p* = 0.0003).

**FIGURE 2:**
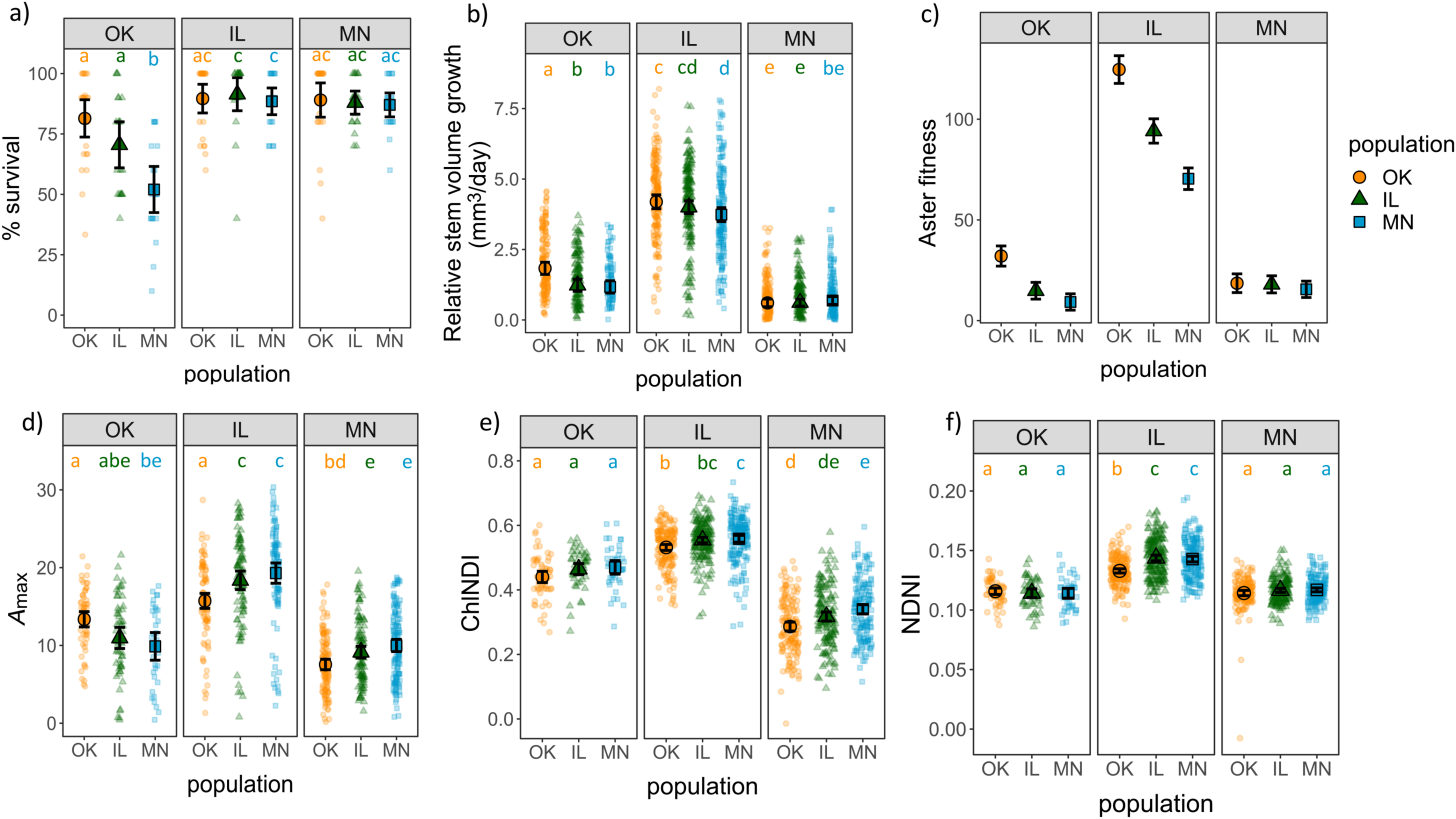
Fitness, fitness components, and physiology in each garden. Each panel represents the common garden located in Oklahoma (OK), Illinois (IL), or Minnesota (MN). Progeny from each source population are represented by orange circles for OK, green triangles for IL, and blue squares for MN, with larger points indicating population means. Distinct letters denote statistical significance (*p* < 0.05) for each population group. Percent survival is represented by a point for each maternal family (a) and was highest for the local OK population in OK, but was similar among all populations in the northern gardens. The relative growth rate of stem volume for individuals (b) was also highest for the local population in OK, with the lowest growth rates occurring in the MN population. Aster expected fitness (c) is represented by a point for each population, and was highest in the IL garden. Aster fitness significance was determined by omnibus tests of population × garden interactions. Maximum CO_2_ assimilation (*A*_max_) (d) of the local populations was highest for the OK and MN garden, but overall the IL garden had the highest *A*_max_. Spectral chlorophyll normalized difference index (ChlNDI) (e) was also higher overall in the IL garden for all populations, though the local MN population was highest in the MN garden. Normalized difference of the nitrogen index (NDNI) (f) indicates that leaf nitrogen was highest in the IL garden.

Stem volume combines the effects of diameter and height growth. There was a significant effect of population on RGR of stem volume (RGR) *F*(2, 1523) = 5.37, *p* = 0.0047. The OK population had the highest stem volume RGR in the Oklahoma garden but was similar to the IL population in the Illinois garden (Figure 2b). Garden also had a strong effect on stem volume RGR (*F*(2, 1523) = 704.46, *p* < 0.0001). In the Minnesota garden, the stem RGR was similar among all populations. Stem volume RGR was highest in the Illinois garden for all populations. In addition, the population × garden interaction was significant for RGR (*F*(4, 1523) = 4.34, *p* = 0.0017).

### Fitness

We found significant (χ^2^(65) = 125.08, *p* = 0.00001) interaction effects of population × garden on fitness using omnibus tests. Block was not a significant predictor of fitness. Our model showed that aster fitness was highest in the Illinois garden for all populations, with the OK population having the highest fitness, in line with our hypothesis of climate change having shifted ranges northward (Figure 2c). In the Oklahoma garden, the OK population had higher fitness than the northern populations, as expected with local versus foreign local adaptation. Fitness in the Minnesota garden was about the same for all populations.

### Physiology

We evaluated photosynthetic responses in each garden. There was a significant population effect for the maximum CO_2_ assimilation (*A*_max_) (*F*(2, 804) = 8.8, *p* = 0.0002). *A*_max_ was highest for the MN and IL populations in the Illinois garden (Figure 2d). Garden had a significant strong effect on *A*_max_ (*F*(2, 804) = 274, *p* < 2e−16). The MN and IL populations had similar *A*_max_ rates to each other in all gardens and were lower in the Oklahoma garden than in the northern gardens, as we hypothesized given the aridity stress in the Oklahoma garden. There was a significant population × garden effect (*F*(4, 804) = 9.7, *p* = 1.2e−7). The OK population had lower *A*_max_ rates in both the Minnesota and Illinois gardens compared to the northern populations but had higher *A*_max_ rates in the Oklahoma garden compared to the MN population.

We found significant effects on ChlNDI with population (*F*(2, 1168) = 22.2, *p* = 3.3e−10) and with garden (*F*(2, 1168) = 1209, *p* < 2e−16), but no significant population × garden interaction effect. Across gardens, the OK population had lower chlorophyll content (as detected by the spectral chlorophyll normalized difference index [ChlNDI]) than the MN population, while IL population values were intermediate. Across populations, ChlNDI was highest in the Illinois garden (Figure 2e). There was a significant population × garden effect (*F*(4, 1168) = 8.1, *p* < 2e−6) on spectral NDNI, and significant effects on NDNI with population (*F*(2, 1168) = 25, *p* = 2.3e−11) and of garden on NDNI (*F*(2, 1168) = 470, *p* < 2e−16). The post hoc Tukey honestly significant difference (HSD) test indicated that the population effect differed in the Illinois garden, where the OK population had lower leaf nitrogen content than the northern populations in the Illinois garden. No significant differences between populations were detected in the Minnesota and Illinois gardens. We found that NDNI was highest for all populations in the Illinois garden, indicating higher leaf nitrogen content (Figure 2f). The NDNI was similar for all populations in the Oklahoma and Minnesota gardens. Data from 2022 were significantly different from 2023 data but indicated similar though weaker trends, which may be an effect of irrigation or ontogeny. We focus on 2023 data here as the effects are stronger and indicate responses in natural conditions without irrigation (see Appendix S1: Figure S4 for 2022 results).

### Relationships between survival, growth, and physiology

Stem volume growth increased significantly (*p* < 0.05) with increases in survival for all populations in the Illinois garden (OK *t* = 2.4, df = 22, *p* = 0.02; IL *t* = 4.3, df = 17, *p* = 0.0004; MN *t* = 2.1, df = 18, *p* = 0.047), but the relationship was not significant in the Minnesota or Oklahoma gardens (Figure 3a). We found that *A*_max_ significantly increased with stem volume growth (*p* < 0.05) for the OK and IL populations in the Oklahoma (OK *t* = 4.1, df = 60, *p* = 0.0001; IL *t* = 3.7, df = 50, *p* = 0.0005), Illinois (OK *t* = 6.7, df = 98, *p* = 1.2e−9; IL *t* = 6.8, df = 94, *p* = 7.9e−10), and Minnesota (OK *t* = 2.5, df = 120, *p* = 0.012; IL *t* = 3.7, df = 109, *p* = 0.0003) gardens, and for the MN population in the Illinois garden (*t* = 6.2, df = 94, *p* = 1.5e−8) (Figure 3b). We also found significant (*p* < 0.05) positive relationships between ChlNDI and stem volume growth in the Illinois garden for all populations (OK *t* = 6.9, df = 176, *p* = 8.8e−11; IL *t* = 5.1, df = 180, *p* = 7.4e−7; MN *t* = 6.4, df = 178, *p* = 1.6e−9), and for the IL and MN populations in the Minnesota garden (IL *t* = 4.4, df = 159, *p* = 1.6e−5; MN *t* = 3.4, df = 162, *p* = 0.00095) (Figure 3c). We did not find significant relationships with survival for *A*_max_ or ChlNDI (not shown).

**FIGURE 3:**
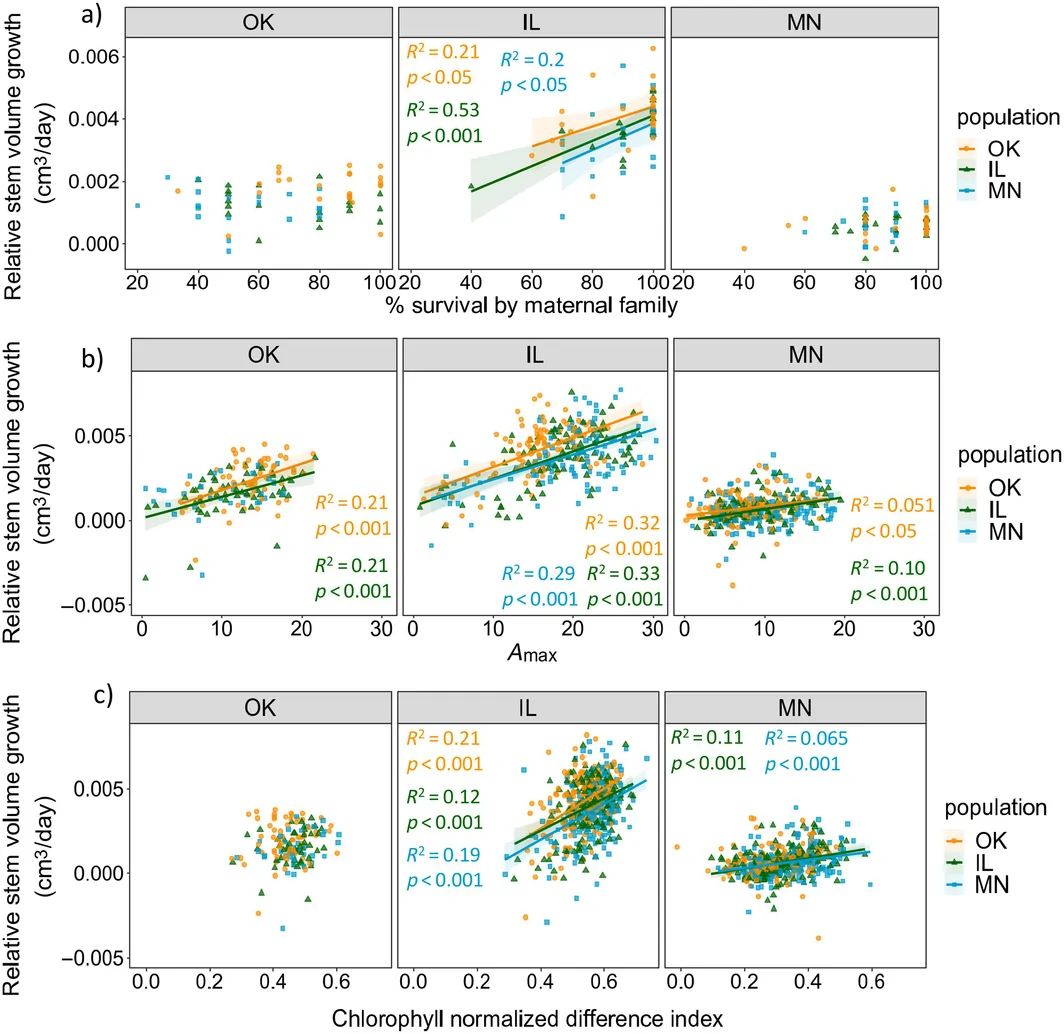
Comparisons of growth, survival, and physiology from the 2023 data collection period to understand shifts in carbon allocation. Each panel represents a common garden located in Oklahoma (OK), Illinois (IL), or Minnesota (MN). The populations are represented by an orange circle for OK, a green triangle for IL, and a blue square for MN, with significant trendlines (with SEs) in matching colors. (a) Percent survival by maternal family is significantly positively correlated with relative stem volume growth for all populations in the Illinois garden, but not in the OK or MN gardens. Maximum photosynthetic rate (*A*_max_) has significant relationships with growth (b) for OK and IL populations in all gardens, and for the MN population in the IL garden. Chlorophyll normalized difference index (ChlNDI) was significantly positively correlated with relative growth rate (c) in IL for all populations, and was significantly correlated with growth in the MN garden for the IL and MN populations.

### Soil quality

Soils in the Oklahoma and Illinois gardens were very similar in carbon (C) and nitrogen (N) concentrations, though concentrations in Oklahoma were a bit higher on average (Table 1). Soils in the Minnesota garden had low percent nitrogen and percent carbon. Minnesota had the highest soil C:N, followed by the Illinois and then the Oklahoma gardens. The soils also differed texturally. Oklahoma garden soils were classified as silty clay loam, Illinois as sandy clay loam, and Minnesota as sandy loam.

**TABLE 1:** Soil properties in each of the three gardens, with variances in parentheses.

Garden	Mean % nitrogen	Mean % carbon	C:N	Soil texture
MN	0.06 (0.0007)	1.04 (0.0757)	21.32	Sandy loam
IL	0.25 (0.0011)	3.54 (0.1584)	14.12	Sandy clay loam
OK	0.30 (0.0035)	3.33 (0.3123)	11.21	Silty clay loam

Abbreviations: IL, Illinois; MN, Minnesota; OK, Oklahoma.

### Environmental gradients across gardens

The gardens fall across two main PCA axes that together account for 100% of the total environmental variance (Figure 4). PC1 accounts for 59.43% of the variance and represents the gradient of temperatures and soil nutrients, with higher values corresponding to higher temperatures and nutrients. PC2 captures 40.57% of the variance and has higher values corresponding to higher precipitation and higher IM (meaning less aridity). The Oklahoma garden falls on the right side of PC1 and the bottom of PC2, indicating that it has high temperatures and nutrients, but low precipitation and high aridity. The Minnesota garden similarly falls on the bottom of the PC2 axis but is to the left of the PC1 axis, indicating it is a dry environment, with lower temperatures and lower nutrients. The Illinois garden lies on the top end of the PC2 axis, indicating high precipitation and low aridity, but falls in the middle of PC1, putting it at intermediate soil nutrients and temperatures.

**FIGURE 4:**
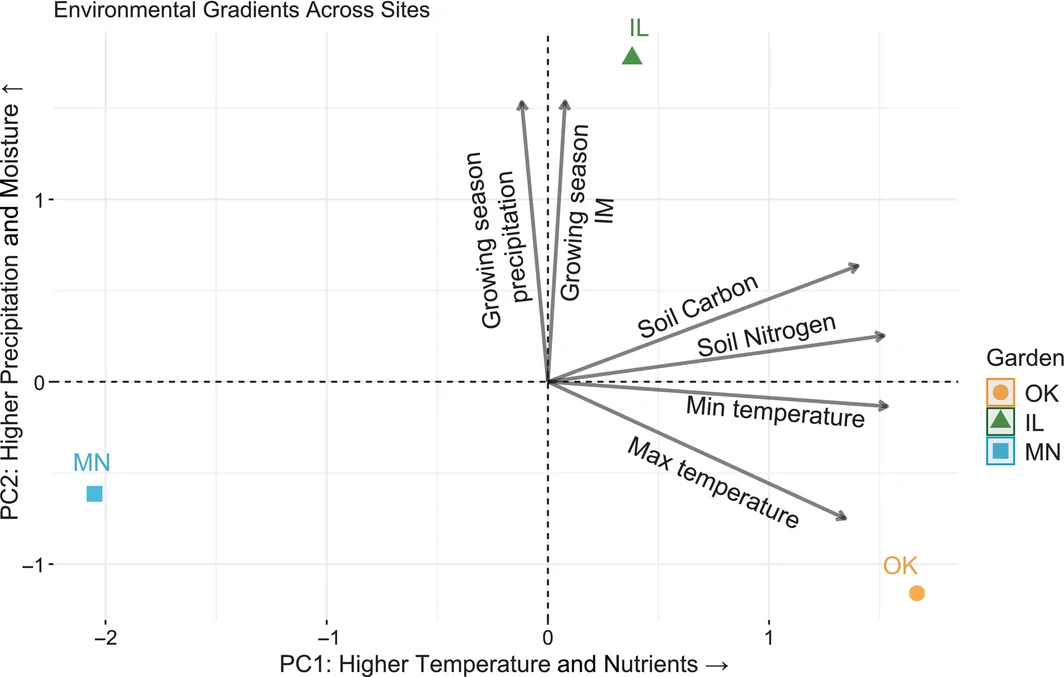
Principal components analysis (PCA) biplot depicting environmental gradients across gardens, with PC1 plotted as the *x* axis and PC2 plotted on the *y* axis. Climate and soil conditions including growing season precipitation, growing season index of moisture (IM), soil carbon, soil nitrogen, minimum (min) temperature, and maximum (max) temperature are indicated by labeled arrows, with the direction indicating where they fall along the PCA axes. The estimates for the Oklahoma (OK), Illinois (IL), and Minnesota (MN) gardens are plotted as points based on where their environmental conditions fall along the gradient between PC1 and PC2. This PCA indicates that temperature and soil nutrients increase with PC1 and precipitation and moisture increase with PC2.

Regressions of performance metrics on PCA axes indicate that PC2 is a consistently strong predictor of performance, indicating that less arid climates lead to better performance and survival (Appendix S1: Table S3). PC2 was a particularly strong predictor for *A*_max_, fitness, and survival. PC1 was positively associated with all traits except survival, indicating that environments with hotter temperatures and higher nutrients lead to lower survival.

### Within and among garden variance in soil and the impact on performance

Soil nitrogen and carbon had varying degrees of significant (*p* < 0.05) effects on mortality and ChlNDI (Table 2). Mortality decreased with increasing soil nitrogen, and increased with increasing soil carbon. Soil nitrogen had a significant decreasing effect on ChlNDI. Being part of the OK population had a significant positive effect on growth rate, but a negative effect on *A*_max_ rates. Both ChlNDI and NDNI had more variance among gardens than within gardens. Survival had greater variance among blocks within gardens than among gardens. RGR had relatively little variance among gardens.

**TABLE 2:** Results of mixed models to evaluate within garden variance.

Trait and effect	Estimate	Variance	df	AIC
Relative growth rate			8	−15,587.3
Fixed effect				
Population IL (intercept)	1.21 × 10^−3^ ± 9.97 × 10^−4^			
Population MN	−1.51 × 10^−4^ ± 9.11 × 10^−5^			
Population OK	2.56 × 10^−4^ ± 8.85 × 10^−5^*			
Soil nitrogen	−1.71 × 10^−3^ ± 5.05 × 10^−3^			
Soil carbon	3.86 × 10^−4^ ± 4.96 × 10^−4^			
Random effect				
Block:Garden		1.259 × 10^−7^		
Garden		1.974 × 10^−6^		
Residual		2.055 × 10^−6^		
Mortality			7	1578.9
Fixed effect				
Population IL (intercept)	1.65 ± 0.39*			
Population MN	−0.52 ± 0.15*			
Population OK	0.23 ± 0.17			
Soil nitrogen	−17.13 ± 4.42*			
Soil carbon	1.38 ± 0.42*			
Random effect				
Block:Garden		2.75 × 10^−1^		
Garden		6.898 × 10^−10^		
Residual				
*A*_max_			8	4886.2
Fixed effect				
Population IL (intercept)	10.8 ± 4.0			
Population MN	0.722 ± 0.42			
Population OK	−1.06 ± 0.41*			
Soil nitrogen	−19.17 ± 24.97			
Soil carbon	2.2 ± 2.37			
Random effect				
Block:Garden		3.128		
Garden		17.118		
Residual		22.823		
ChlNDI			8	−2682.1
Fixed effect				
Population IL (intercept)	4.3 × 10^−1^ ± 7.22 × 10^−2^*			
Population MN	1.15 × 10^−2^ ± 5.38 × 10^−3^*			
Population OK	2.67 × 10^−2^ ± 5.35 × 10^−3^*			
Soil nitrogen	−4.88 × 10^−1^ ± 2.04 × 10^−1^*			
Soil carbon	4.34 × 10^−2^ ± 1.98 × 10^−2^			
Random effect				
Block:Garden		1.579 × 10^−4^		
Garden		1.135 × 10^−2^		
Residual		5.628 × 10^−3^		
NDNI			8	−6707.4
Fixed effect				
Population IL (intercept)	1.21 × 10^−1^ ± 9.42 × 10^−3^*			
Population MN	−2.36 × 10^−4^ ± 9.66 × 10^−4^			
Population OK	−5.58 × 10^−3^ ± 9.6 × 10^−4^*			
Soil nitrogen	2.26 × 10^−2^ ± 3.66 × 10^−2^			
Soil carbon	−9.22 × 10^−5^ ± 3.54 × 10^−3^			
Random effect				
Block:Garden		5.086 × 10^−6^		
Garden		1.996 × 10^−4^		
Residual		1.817 × 10^−4^		

*Note*: SE is included with the estimates of fixed effects, and significance of estimates at the *p* < 0.05 level is indicated with an asterisk (*).

Abbreviations: AIC, Akaike information criterion; ChlNDI, chlorophyll normalized difference index; IL, Illinois; MN, Minnesota; NDNI, normalized difference nitrogen index; OK, Oklahoma.

### Phenology and growing season

Budburst was recorded earliest in the Oklahoma garden and latest in the Minnesota garden, on average. We found significant effects of budburst with population *F*(2, 984) = 78.6, *p* < 2.2e−16, garden *F*(2, 984) = 508.8, *p* < 2.2e−16, and a significant interaction of population × garden *F*(4, 984) = 16.3, *p* = 6.2e−13. Of the three populations, the OK population tended to break bud earliest on average, except in the Minnesota garden where the MN population broke bud slightly earlier, though there was no significant difference in budbreak among populations in the Minnesota garden (Figure 5a). We found significant effects on fall senescence with population *F*(2, 984) = 33, *p* < 2.2e−16, garden *F*(2, 984) = 218.5, *p* < 2.2e−16, and a small but significant interaction of population × garden *F*(4, 984) = 2.9, *p* = 0.02. The Minnesota garden had the earliest date of senescence, whereas the Oklahoma garden was the latest (Figure 5b). The OK population also tended to senesce later than the northern populations, particularly in the Illinois garden. The Oklahoma garden had the longest growing season length for all three populations, whereas Minnesota was the shortest (Figure 5c).

**FIGURE 5:**
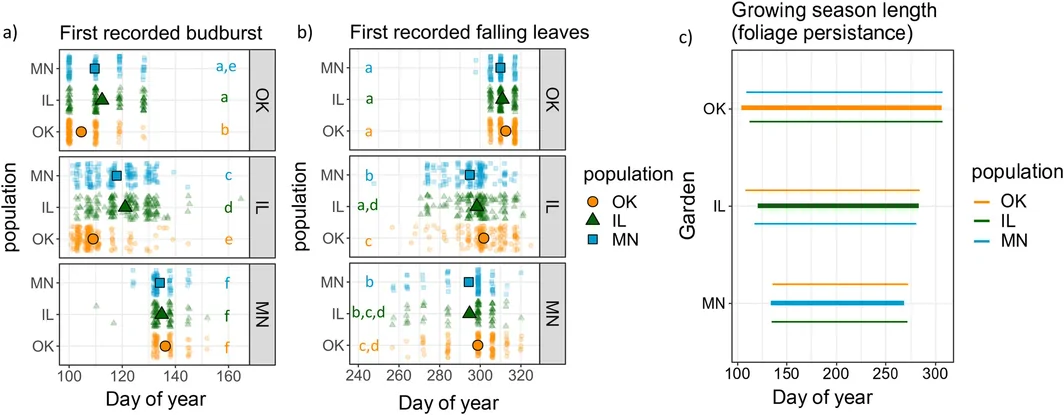
Phenology and growing season length in each of the gardens with data collected in 2023. Each panel represents a common garden located in Oklahoma (OK), Illinois (IL), or Minnesota (MN). The populations are represented by an orange circle for OK, a green triangle for IL, and a blue square for MN. Budburst (a) occurs earlier in the OK and IL garden for all populations than in the MN garden, and senescence (b) occurs latest in the OK garden. The growing season length (c) is shortest in the MN garden and longest in the OK garden.

## DISCUSSION

### Evidence for local adaptation

We established the ACE experiment, a reciprocal transplant experiment along a latitudinal gradient, to test for local adaptation and adaptive differentiation among populations of *Q. macrocarpa* from contrasting climates and environments. For all populations, we expected that local adaptation would be demonstrated by higher fitness of the local population relative to other populations (local vs. foreign) and higher fitness of each population in its home location relative to its fitness in other locations (home vs. away). We found evidence supporting local adaptation based on the local versus foreign comparison in the Oklahoma garden with the hottest climate. The OK source population had higher survival, growth, and fitness in the Oklahoma garden compared to the other populations, which supports local versus foreign adaptation.

Under the 2°C warming expected (IPCC, 2023) with climate change, the OK population may be better suited for the emerging warmer climates with potential to expand northward. These results can inform ongoing efforts to manage forests and identify seed collection zones for transferring seeds among regions by demonstrating that the southernmost population is adapted to warmer climates. Populations adapted to warmer climates may be suitable for reforestation efforts aimed at improving forest resilience to climate change or transitioning ecosystems to novel climates (Palik et al., 2022). In addition, the lack of evidence for home versus away local adaptation for the OK population in colder, northern climates further supports the transfer of southern seed northward, as supported by the lack of population differentiation for cold hardiness in *Larix occidentalis* (Roskilly & Aitken, 2024).

Despite evidence of local adaptation in the Oklahoma garden, our results show that all populations expressed highest fitness in the Illinois garden. Consequently, we did not find evidence for local adaptation based on home versus away comparisons. This effect may be due in part to differences in management—the gardens were watered on differing schedules (Appendix S1: Section S3, Methods supplement), and the Illinois garden was also weeded more intensively than the other two gardens—but the effect is not entirely or, we believe, even primarily due to management—given the differences between the three gardens in precipitation, extremes of temperature, and soils (Figures 1b–d and 4).

We also did not find clear statistical evidence for local versus foreign local adaptation in the two northern populations in their respective gardens after 3 years of growth. However, these patterns of local adaptation could still emerge through time or could be obscured by maternal effects. The OK population had a higher absolute growth rate, which could be due in part to its larger acorn size (Appendix S1: Table S4) that supports growth and survival in its early years (Bischoff et al., 2006; Deacon & Cavender‐Bares, 2015). We were not able to measure all critical ontogenetic stages and lifetime components of fitness, including germination and fecundity. Further studies in the Illinois garden have demonstrated that height and flowering are positively correlated, yet the OK population (which tends to be the tallest) had the fewest flowering individuals (Mohn et al., 2026). Reproductively mature life stages in other gardens could still reveal evidence of local adaptation not evident at the seedling life stage (Wadgymar et al., 2022). Nevertheless, the patterns of maladaptation to warmer climates we observe in northern populations could also increase in later life stages (Goetz et al., 2024, 2025). It is critical to study seedling survival and function to understand the future of tree populations, given that seedlings often have a narrower climatic niche and are more vulnerable compared to adult trees, and the critical role of seedling establishment to the regeneration and persistence of tree populations (Davis et al., 1998; Dobrowski et al., 2015; Williams, 2021).

Our alternative hypothesis for the observation of high performance beyond a population's local garden was that ongoing climate change has shifted suitable ranges of all populations northward, leading southern populations to be better adapted to northern gardens. However, while we saw that the OK population did perform better in the Illinois garden, which could be a consequence of both edaphic and climatic factors, we did not see evidence that the IL population performed better in the Minnesota garden compared to in its home garden, nor did it outperform the local MN population in Minnesota. This result indicates that there may be additional conditions or stressors in the Minnesota garden, such as freezing, nutrient deficiencies, and/or insect herbivory, that influence the performance of nonlocal populations.

### Variation in season length helps explain carbon assimilation and growth patterns

In other provenance trials performed with conifer species, populations from warmer climates are associated with higher growth rates and tend to decrease in growth rates as the transfer distance from their home climate increases (Genys, 1987; Li et al., 2020; Zhuk & Goroshkevich, 2018). Higher survival, growth rates, and fitness in the Illinois garden for all populations were likely a consequence of productive soil conditions and mild climate relative to more stressful environments (the cold of Minnesota, the aridity of Oklahoma) found closer to the margins of *Q. macrocarpa*'s range. Better survival, growth, and fitness in milder climates aligns with similar findings in *Pinus albicaulis* from milder climates in limited stress (Warwell & Shaw, 2017). Aridity stress in the Oklahoma garden led to lower photosynthetic rates in northern populations, as evidenced by the association between moisture levels and *A*_max_ rates (Table 2). The decline in assimilation rates in seedlings from the IL and MN populations in the hotter and drier Oklahoma climate suggests that these populations may experience physiological stress leading to future declines in survival and fitness in their current habitats. The OK population, given that it originates from a more arid climate, may have better survival in response to drought (Warwell & Shaw, 2017).

In addition, the OK population had the highest maximum carbon assimilation rate (*A*_max_) of the three populations in the Oklahoma garden. The lower *A*_max_ rate of the northern populations in the Oklahoma garden indicates they were stressed in the hotter climate. Drought is known to reduce stomatal conductance, which limits carbon assimilation (Boyer, 1976), and the Oklahoma garden was also the most arid. Previous findings from oaks have shown that *A*_max_ is reduced when plants are exposed to chronic drought (Gimeno et al., 2008).

Differences in growing season lengths along the latitudinal gradient also played a role in the populations' photosynthetic and growth responses. Fall senescence in *Quercus rubra* is less responsive to warming temperature over time, but spring leaf out can shift earlier with warming (Knott et al., 2023), which may impact transplanted populations. Later fall senescence may have caused dieback of stems (negative growth) due to frost damage of the OK population in the Minnesota garden, consistent with previous work in another North American temperate oak species (*Q. rubra*) that implicates autumn frost damage at northern range limits growth of trees in southern seed sources (Lindback et al., 2023). The OK population broke bud earlier and senesced later in the Oklahoma and Illinois gardens relative to other populations, consistent with findings in other oak common gardens (Papper & Ackerly, 2021; Wright et al., 2021), which supports our expectations of populations from lower latitudes being adapted to longer growing seasons.

The Oklahoma population tended to maintain a relatively low rate of carbon assimilation across gardens; compared to the other populations in the northern gardens, it had the lowest carbon assimilation rate. This is in accordance with expectations for slow life history strategies, where leaves with longer life spans are theoretically expected and empirically demonstrated to have slower rates of photosynthesis (Chabot & Hicks, 1982; Wright et al., 2004). On the other hand, the northern populations that are adapted to shorter growing seasons tended to have higher *A*_max_ rates than the Oklahoma population in the northern gardens. This trend of higher photosynthetic rates is consistent with findings in a *Betula pendula* provenance trial in which northern populations had greater net photosynthesis (Tenkanen et al., 2021) and has implications for the growth and survival of northern populations as climate change extends growing season length. These stressors of heat (Oklahoma) and frost (Minnesota) in the gardens at the ends of our latitudinal gradient likely explain the high performance of seedlings in the milder Illinois garden.

Stressful conditions can also shift carbon allocation away from growth toward drought stress responses and defense (Wang et al., 2024). We found correlations between growth and survival in the Illinois garden, in which all populations expressed highest fitness, likely due to its relatively mild climate and loamy soils. Previous work in the European pedunculate oak, *Quercus robur*, indicates that differences in soil conditions and water availability across sites with similar climate regimes lead to variation in productivity (Buras et al., 2020). Growth and survival were decoupled in both the Oklahoma and Minnesota gardens, which may be a consequence of resource allocation trade‐offs under increased stress (heat in Oklahoma, cold and low nutrient availability in Minnesota, and aridity in both) that improve survival at the expense of growth rates (Bazzaz et al., 1987; Willi & Van Buskirk, 2022). In addition, despite having higher soil nitrogen levels, the fine‐textured silty clay soils in Oklahoma may be less conducive to nutrient access.

Carbon assimilation and light energy acquisition through photosynthesis were important for growth, as evidenced by the positive correlations between growth and *A*_max_ and leaf chlorophyll content, particularly in Illinois. In fertile sites, RGRs and photosynthetic rates tend to be higher, as was the case in the Illinois garden, where conditions were favorable (Lambers & Poorter, 1992). In the Illinois garden, the seedlings were able to allocate most of their carbon toward aboveground growth, which in turn improved survival, similar to findings of a lack of trade‐off in growth and survival with climate transfer in *Picea glauca* (Benomar et al., 2016). On the other hand, in the less favorable, more arid conditions of Minnesota and Oklahoma, survival likely depended on allocating carbon elsewhere, such as drought adaptation, root growth, or defense against herbivory (Coley et al., 1985; Poorter et al., 2012). Selecting populations that are able to shift allocation of carbon to improve survival will likely be important given the expected increased intensity of drought and pests with climate change.

### Multiple environmental factors vary across gardens

An outdoor common garden experiment such as ours, with unmanipulated soils, cannot isolate climate as the sole factor that varies among sites, and it may well turn out that the seedlings in the Illinois garden perform best due to non‐climatic factors. Edaphic factors, including unmeasured factors such as the microbial environment, vary among and within experimental replicates (in three states). We found that the environmental conditions in gardens fell along a gradient of soil nutrients and temperature and a gradient of aridity and precipitation. Total soil nitrogen concentration was similar between the Illinois and Oklahoma gardens, but the less arid and slightly cooler climate in the Illinois garden combined with the high soil nitrogen and soil organic matter of the loamy soil created an environment that is conducive to bur oak carbon assimilation and fitness, as was shown with the decrease in mortality with greater nitrogen. We also note that of the three gardens, Illinois had the least change in growing season temperature since 1940 (Appendix S1: Table S2). The fertile soil and relatively stable, mild climate of the region near the Illinois garden may provide important habitat for bur oak populations to grow with ongoing climate change.

In addition, differences in populations' growth and phenological timing could be due to a lack of chilling fulfillment or accumulation of growing degree days, as well as differences in the timing of daylight across the gradient (Clark et al., 2014; Taulavuori et al., 2017; Vitasse et al., 2011). Controlled environment experiments that isolate climate as the driving variable for performance changes in bur oaks are an important next step in predicting the response of bur oak populations to climate change, following experiments such as (Browne et al., 2019; Sáenz‐Romero et al., 2017), which have tested climate response in other oak species in Mediterranean climates, which have found evidence of adaptational lag and negative responses of trees to warmer climates.

## CONCLUSION

Findings from the ACE bur oak reciprocal transplant experiment provide context for seed transfer aimed at improving resilience of northern tree populations in the face of climate change. Our results indicate that northern bur oak populations may have high mortality under high levels of warming with climate change. We found evidence that the northern population was poorly adapted to the hottest garden's conditions in the south, and populations originating from warmer southern climates perform well even when they are planted northward into Illinois. These findings indicate that southern locations may provide valuable seed sources to augment existing genetic variation in northern regions in the face of ongoing climatic warming. Consideration of current climate and environmental conditions alongside future climate conditions is important in decisions to move locally adapted populations. It may be beneficial to ensure seeds are sourced from locations with varied climates to accommodate fluctuations in climate. It is also worth noting that the expected change in climate may be within the tolerance limits of existing populations in the north. Mild climate warming akin to the temperature difference between the Minnesota and Illinois gardens in the case of *Q. macrocarpa*, as demonstrated by improved performance and fitness for the MN populations in the Illinois garden. However, the poor performance of the MN population in the warmest garden may need to be considered in long‐term forest planning, given the rapid rates of warming in northern latitudes, and may require seed transfer from southern seed sources to supplement and maintain northern bur oak populations and advance the rate of adaptation to warmer climates. We did not test performance under competition with other species nor explicitly vary edaphic environments across climates; however, the performance of oaks across contrasting edaphic environments in competition with other tree species is an important area of investigation for future studies.

## AUTHOR CONTRIBUTIONS

All authors contributed intellectually to the study. Jeannine Cavender‐Bares designed the ACE experiment and managed its implementation. Jeannine Cavender‐Bares, Andrew L. Hipp, and Heather R. McCarthy managed the collaboration and secured funding for the study. Lucy M. S. Rea led data collection, analysis, and interpretation, and writing of the manuscript. Jeannine Cavender‐Bares led nursery planting and contributed to data interpretation. Cathleen Lapadat, Lindsey Worcester, and Mira Garner led planting efforts in the gardens. Laura Ostrowsky, Rebekah Mohn, Mira Garner, Lindsey Worcester, and Cathleen Lapadat collected data. Jeannine Cavender‐Bares, Andrew L. Hipp, and Heather R. McCarthy edited early versions of the manuscript. All authors provided critical revision of the article and provided final approval of the version to be published.

## CONFLICT OF INTEREST STATEMENT

The authors declare no conflicts of interest.

## Supporting information


Appendix S1.

## Data Availability

Data and code (Rea et al., 2026a) are available in Dryad at https://doi.org/10.5061/dryad.2z34tmpvk.
