# Supplementary material for: Variation among populations of Quercus macrocarpa in a reciprocal transplant experiment across a latitudinal gradient
Source: Ecology. 2026 Oct 4;107(10):e70517. doi: 10.1002/ecy.70517 (PMC13635612; doi:10.1002/ecy.70517)
Supplement: Supplementary file 1 [file ECY-107-e70517-s001.pdf]

Appendix S1

Variation among populations of *Quercus macrocarpa* in a reciprocal transplant experiment across a latitudinal gradient

Lucy M.S. Rea, Laura Ostrowsky, Rebekah Mohn, Mira Garner, Lindsey Worcester, Cathleen Lapadat, Heather R. McCarthy, Andrew L. Hipp, Jeannine Cavender-Bares

Ecology

Section S1: Figures

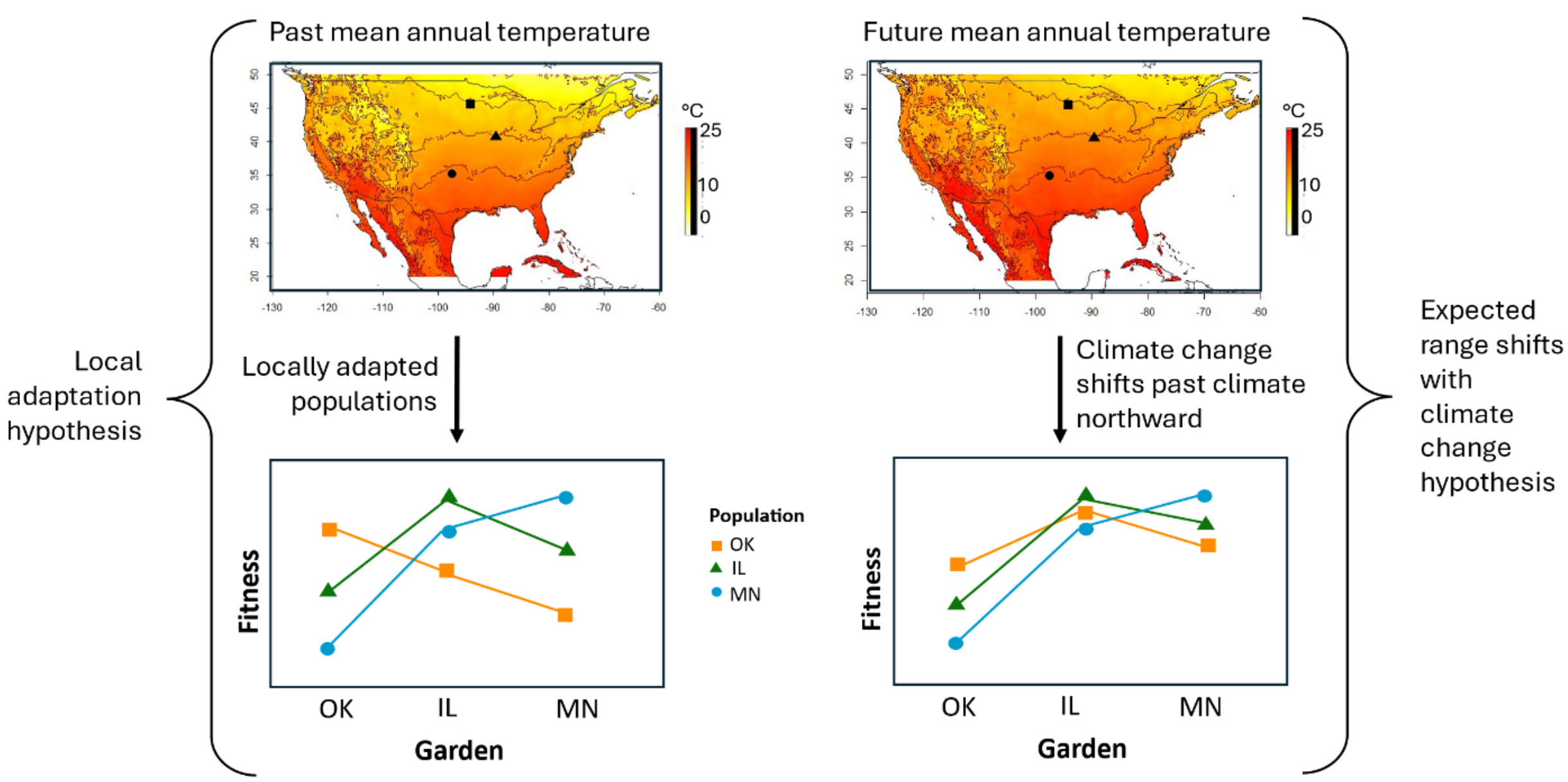

Figure S1. Illustrated hypotheses for local adaptation, adaptive differentiation, maladaptation, and shifting habitat suitability with climate change. The left panels represent classic local adaptation predictions where home populations have higher fitness than away populations with significant differences in fitness among populations in each garden, and each population has the highest fitness in its local garden. Populations have lower fitness in away gardens under the classic local adaptation hypothesis, indicating maladaptation. The right panels illustrate expectations under a warming climate, where populations still have higher fitness in their home garden compared to the away populations, but tend to have higher fitness in northern gardens compared to southern gardens due to shifting climate. These hypotheses do not take into account other environmental factors (including edaphic and microbial factors) that may be relevant to local adaptation.

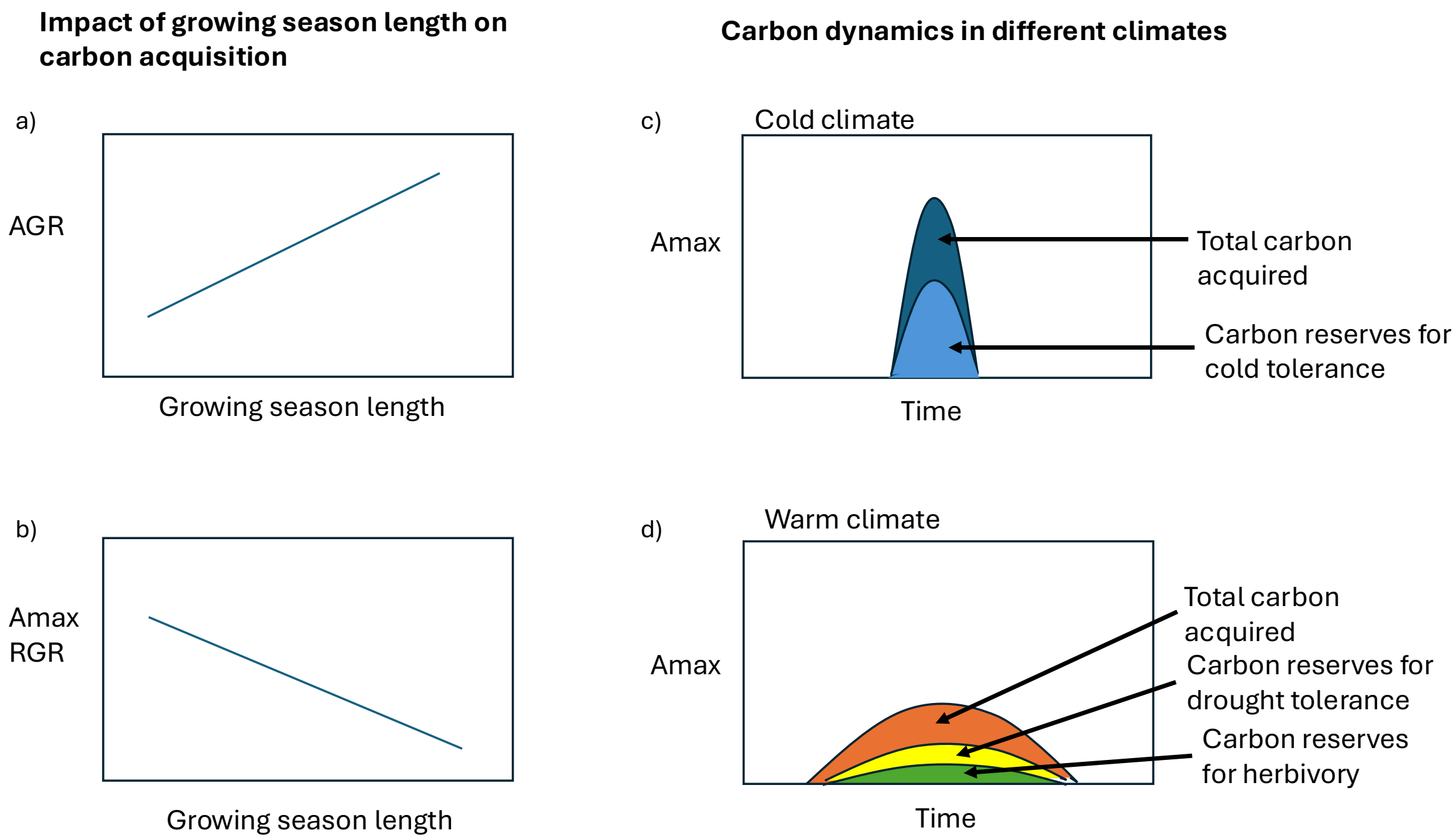

Figure S2. Predicted associations of growing season length with (a) absolute growth rate (AGR) and carbon assimilation rate (A_{max}) and relative growth rate (b). Total growth over a growing season is expected to increase with a longer growing season, however, with longer growing seasons, the carbon assimilation rate and relative growth rate are expected to be slower and spread out throughout the longer growing season. We expect that carbon allocation will shift due to stressors introduced in colder (a) or warmer (b) climates. In cold climates, carbon acquisition is expected to occur at a fast rate over a short period of time allowed by the growing season. A portion of that carbon acquired is expected to be allocated toward non structural carbon reserves for cold tolerance. In warmer climates, carbon acquisition is expected to occur over a prolonged period over a slower rate. A portion of the carbon may be allocated toward drought tolerance or toward herbivore defense. The remaining carbon is available for allocation toward growth.

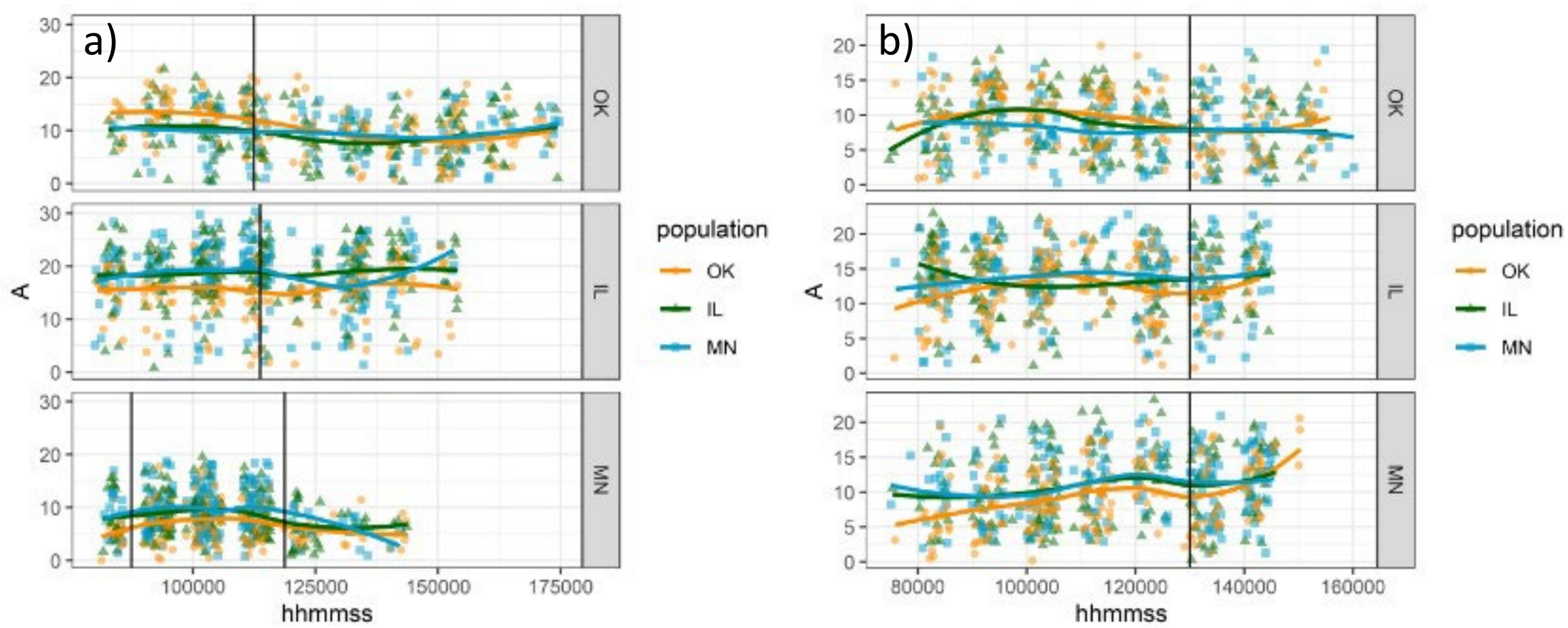

Figure S3. Diurnal curves for photosynthetic carbon assimilation (A) in each garden, with time of day on the x axis in 2023 (a) and 2022 (b). Each panel represents a garden, and each point represents an individual measurement. Black vertical lines indicate the cutoff points for A_{max}. Populations are represented as orange circles for Oklahoma, green triangles for Illinois, and blue squares for MN. Colored lines indicate the trend in A for each population.

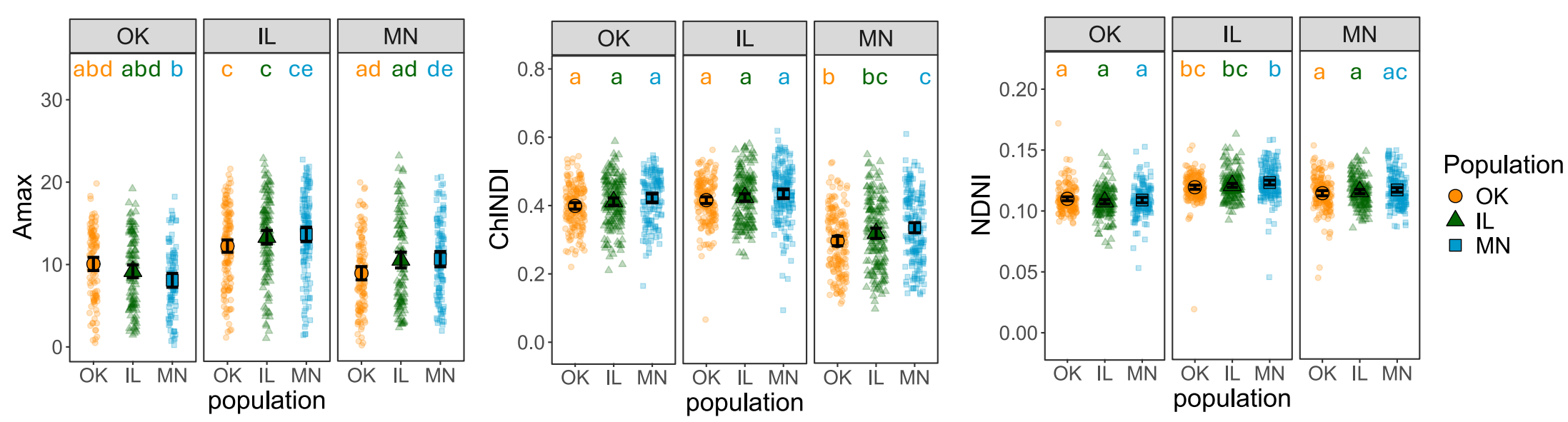

Figure S4. Physiological data from 2022 including the max photosynthetic rates (A_{max}), chlorophyll normalized difference index (ChlNDI), and normalized difference nitrogen index (NDNI). Each panel represents a common garden located in Oklahoma (OK), Illinois (IL), or Minnesota (MN). The populations are represented by an orange circle for OK, a green triangle for IL, and a blue square for MN. Distinct letters denote statistical significance ($p<0.05$) for each population group.

Section S2: Tables

Table S1. Number of individuals for each measure for each population in each garden.

Measure	Garden	Population	Number of individuals
A_{max}	OK	OK	62
A_{max}	OK	IL	57
A_{max}	OK	MN	34
A_{max}	IL	OK	101
A_{max}	IL	IL	96
A_{max}	IL	MN	96
A_{max}	MN	OK	123
A_{max}	MN	IL	112
A_{max}	MN	MN	132
ChlNDI	OK	OK	61
ChlNDI	OK	IL	48
ChlNDI	OK	MN	42
ChlNDI	IL	OK	179
ChlNDI	IL	IL	183
ChlNDI	IL	MN	180
ChlNDI	MN	OK	157
ChlNDI	MN	IL	161
ChlNDI	MN	MN	165
gswMAX	OK	OK	62
gswMAX	OK	IL	57
gswMAX	OK	MN	34
gswMAX	IL	OK	101
gswMAX	IL	IL	96
gswMAX	IL	MN	96

gswMAX	MN	OK	123
gswMAX	MN	IL	112
gswMAX	MN	MN	132
$iWUE_{max}$	OK	OK	62
$iWUE_{max}$	OK	IL	57
$iWUE_{max}$	OK	MN	34
$iWUE_{max}$	IL	OK	101
$iWUE_{max}$	IL	IL	96
$iWUE_{max}$	IL	MN	96
$iWUE_{max}$	MN	OK	123
$iWUE_{max}$	MN	IL	112
$iWUE_{max}$	MN	MN	132
NDNI	OK	OK	61
NDNI	OK	IL	48
NDNI	OK	MN	42
NDNI	IL	OK	179
NDNI	IL	IL	183
NDNI	IL	MN	180
NDNI	MN	OK	157
NDNI	MN	IL	161
NDNI	MN	MN	165
RGR stem volume	OK	OK	167
RGR stem volume	OK	IL	140
RGR stem volume	OK	MN	103
RGR stem volume	IL	OK	185

RGR stem volume	IL	IL	189
RGR stem volume	IL	MN	194
RGR stem volume	MN	OK	184
RGR stem volume	MN	IL	188
RGR stem volume	MN	MN	182

Table S2. Climate from 1940-2023 in each of the three gardens.

**Garden**	**Mean annual temperature (°C) 1940-1970**	**Mean annual temperature (°C) 1993-2023**	**Δ mean annual temperature (°C) 1940-70 to 1993-2023**	**Growing season temperature (°C) 1940-1970**	**Growing season temperature (°C) 1993-2023**	**Δ growing season temperature (°C) 1940-70 to 1993-2023**
Oklahoma	15.61	16.51	**0.91**	26.77	27.54	**0.77**
Illinois	10.28	11.44	**1.16**	22.69	23.41	**0.72**
Minnesota	5.68	6.91	**1.24**	20.14	21.07	**0.92**

Table S3 (following page). Results of regressions of PCA axes with performance metrics of photosynthetic rate (A_{max}), fitness, chlorophyll normalized difference spectral index (ChlNDI), leaf nitrogen normalized difference index (NDNI), survival, and relative growth rate. R^2 values are reported for all but survival, where the McFadden pseudo- R^2 is reported to compare the log likelihood of the full GLM model to the log likelihood of the null model.

Response Variable	Model Type	PC1 Effect	PC2 Effect	Degrees of freedom	R^2 / Pseudo-R^2
**A_{max}**	Linear	+1.13 ($p<2.2x10^{-16}$, t=9.2)	**+2.56** ($p<2.2x10^{-16}$, t=17.3)	810	R^2=0.386
**Fitness**	Linear	**+5.75** ($p<2.2x10^{-16}$, t=14.7)	**+2**8.97 ($p<2.2x10^{-16}$, t=61.3)	1788	R^2=0.69
**ChlNDI**	Linear	**+0.045** ($p<2.2x10^{-16}$, t=25.2)	**+0.051** ($p<2.2x10^{-16}$, t=25.8)	1174	R^2=0.662
**NDNI**	Linear	**+0.001** (p=0.0017, t=3.2)	**+0.009** ($p< 2.2x10^{-16}$, t=254)	1174	R^2=0.431
**Survival**	Logistic	**–0.26** ($p=3.7x10^{-10}$, z=-6.27)	**+0.36** ($p=8.1x10^{-11}$, z=6.5)	1798	McFadden=0.06
**Relative Growth Rate**	Linear	**+0.00037** (p< 2.2x10-16, t=15)	**+0.00094** (p< 2.2x10-16, t=32)	1529	R^2=0.473

Table S4. Average acorn volumes for each of the three populations.

**Population**	**Average acorn volume (cm^3)**
OK	14.7
IL	3.5
MN	2.0

Section S3: Methods supplement

Acorn processing

The acorns were processed by removing the caps and placing them in water, discarding acorns that floated as they were likely to be inviable. Viable acorns were placed in sand in cold storage until planting in the fall.

Garden maintenance

Mulching

In Illinois and Minnesota, the gardens were mulched at the end of the first growing season. In Oklahoma, mulching occurred in 2023 due to challenges sourcing mulching materials.

Irrigation

In Minnesota, for the first year after planting, the seedlings were watered five days a week for eight weeks, then two to three days a week for another four to five weeks. The second year, seedlings were irrigated once a week through July and August. Each time they were irrigated the seedlings were provided approximately 5 mm of water.

In Illinois, for the first two years of planting, the seedlings were watered once a week for 1 hour when there had been no rain. If less than an inch of rain fell, the seedlings were supplemented with water to reach approximately 1 inch of water for the week (approximating that if 0.5 inch of rain fell, the seedlings were watered for 30 mins.; if 0.75 inches of rain fell, the seedlings were watered for 15 mins.). If 1 inch of rain fell, the seedlings were not watered.

Similarly, in Oklahoma for the first two years of planting, the seedlings were watered twice a week for 30 minutes (approximately 1 inch per week total) any time there was not significant rain (>1 inch).

Gas exchange parameters

To keep the measurement conditions consistent across the three gardens, the chamber environment was maintained at 30 °C, with 60% relative humidity and 400 µmol/mol for the reference CO_2. Light was set at PAR of 1200 µmol m^{-2} s^{-1}, as was determined to be saturating based on light curves conducted in the Oklahoma garden on a subset of trees.

Spectral data information and processing

Our spectral measurements encompassed the wavelength range 340 to 2,500 nm with 1,024 spectral bands. The spectral sensors were matched by splicing at 990 nm and 1100 nm and interpolating 5 nm and 1 nm, respectively, using the match_sensors function in *spectrolab* package v. 0.0.18 (Meireles et al. 2021) in R v.4.3.0 (R Core Team 2023).

References

Meireles, JoseEduardo, Anna K. Schweiger, and Jeannine Cavender-Bares. 2021. *Package ' Spectrolab .'*

R Core Team. 2023. *R: A Language and Environment for Statistical Computing*. R Foundation for Statistical Computing, released.
